# Fractionated radiotherapy adjuvant to surgery of WHO-2 meningioma with and without gross total resection: a multicenter, retrospective cohort study of 1,452 patients

**DOI:** 10.1007/s11060-025-05349-7

**Published:** 2026-02-09

**Authors:** Christian Mirian, Lasse Rehné Jensen, Adam Gorm Hoffmann, Tareq A. Juratli, Andrea Daniela Maier, Pernilla Lindner, Anders Broechner, Sverre H. Torp, Helen A. Shih, Ramin A. Morshed, Jacob S. Young, Stephen T. Magill, Walter Stummer, Dorothee Cäcilia Spille, Benjamin Brokinkel, Martin Proescholdt, Yasuhiro Kuroi, Konstantinos Gousias, Matthias Simon, Ricardo Prat-Acin, Stéphane Goutagny, Johannes Wach, Erdem Güresir, Junkoh Yamamoto, Young Zoon Kim, Joo Ho Lee, Daniel W. Kim, Matthew Koshy, Donald M. Cannon, Dennis C. Shrieve, Chang-Ok Suh, Jong Hee Chang, Maria Kamenova, Sven Straumann, Jehuda Soleman, Ilker Y. Eyüpoglu, Tony Catalan, Austin Lui, Philip V. Theodosopoulos, Michael W. McDermott, Pedro Góes, Fang Wang, Luis Souhami, Marie-Christine Guiot, Tamás Csonka, Toshiki Endo, Tejpal Gupta, Akash J. Patel, Tiemo J. Klisch, Jun Won Kim, Francesco Maiuri, Valeria Barresi, María Dolores Tabernero, Simon Skyrman, Mechthild Krause, Ian Law, Bjarne Winther Kristensen, Tina Nørgaard Munch, Torstein Meling, Kåre Fugleholm, Paul Blanche, Tiit Mathiesen

**Affiliations:** 1 Department of Neurosurgery, Copenhagen University Hospital, Copenhagen, Denmark; 2 Section of Biostatistics, Department of Public Health, University of Copenhagen, Copenhagen, Denmark; 3 Department of Neurosurgery, Division of Neuro-OncologyFaculty of Medicine and University Hospital Carl Gustav Carus, Technische Universität Dresden, 01307 Dresden, Germany; 4 Department of Neurosurgery, Laboratory of Translational Neuro-Oncology, Massachusetts General Hospital Cancer Center, Harvard Medical School, Boston, USA; 5 Department of Pathology, Bartholin Institute, Rigshospitalet, Copenhagen University Hospital, Copenhagen, Denmark; 6 Department of Clinical and Molecular Medicine, Faculty of Medicine and Health Sciences, Norwegian, University of Science and Technology (NTNU), Laboratory Centre, St. Olavs Hospital, Trondheim, NO-7491 Norway; 7 Department of Pathology, Laboratory Centre, St. Olavs Hospital, Trondheim, NO-7030 Norway; 8 Department of Radiation Oncology, Massachusetts General Hospital, Harvard Medical School, Boston, MA USA; 9 Department of Neurological Surgery, University of California San Francisco, CA, USA; 10 Department of Neurological Surgery, Northwestern University, Feinberg School of Medicine, Chicago, IL, USA; 11 Department of Neurosurgery, University of Münster, Münster, Germany; 12 Institute for Neuropathology, University of Münster, Münster, Germany; 13 Deparment of Neurosurgery, Clemenshospital, Münster, Münster, Germany; 14 Department of Neurosurgery, University Regensburg Medical Center, Regensburg, Germany; 15 Department of Neurosurgery, Tokyo Women’s Medical University, Adachi Medical Center, Tokyo, Japan; 16 Department of Neurosurgery, Athens Medical Center, Athens, Greece; 17 European University of Cyprus, Nicosia, Cyprus; 18 Department of Neurosurgery, Bethel Clinic University of Bielefeld Medical Center, Bielefeld, Germany; 19 Department of Neurosurgery, Hospital La Fe, Valencia, Spain; 20 Université Paris Cité, Department of Neurosurgery, Beaujon Hospital, Assistance Publique Hôpitaux de Paris, Paris, France; 21 Department of Neurosurgery, University Hospital Leipzig, Leipzig, Germany; 22 Department of Neurosurgery, University of Occupational and Environmental Health, Kitakyushu, Japan; 23 Department of Neurosurgery, Samsung Changwon Hospital, Sungkyunkwan University School of Medicine, Changwon, Republic of Korea; 24 Department of Radiation Oncology, Seoul National University College of Medicine, Seoul National University Hospital, Seoul, Republic of Korea; 25 Cancer Research Institute, Seoul National University College of Medicine, Seoul, South Korea; 26 Department of Radiation Oncology, University of Illinois Hospital and Health Sciences System, Chicago, IL, USA; 27 Department of Radiation Oncology Spencer Fox Eccles School of Medicine University of Utah, Salt Lake City, UT, USA; 28 Department of Radiation Oncology, Yonsei University College of Medicine, Seoul, Republic of Korea; 29 Department of Neurosurgery, Yonsei University College of Medicine, Seoul, Republic of Korea; 30 Department of Neurosurgery, University Hospital Basel, Basel, Switzerland; 31 Division of Neurosurgery, Miami Neuroscience Institute, Miami, FL, USA; 32 Department of Neurosurgery, Federal University of São Paulo, São Paulo, Brazil; 33 Department of Neurosurgery, The First Affiliated Hospital of Zhengzhou University, Zhengzhou, Henan China; 34 Division of Radiation Oncology, McGill University Health Centre, McGill University, Montreal, QC, Canada; 35 Department of Pathology, McGill University Health Centre, Montreal, QC, Canada; 36 Department of Pathology, Faculty of Medicine, University of Debrecen, Debrecen, Hungary; 37 Division of Neurosurgery, Tohoku Medical and Pharmaceutical University, Tohoku, Japan; 38 Department of Radiation Oncology ACTREC, Tata Memorial Centre, HBNI Kharghar, Navi Mumbai, 410210 India; 39 Department of Neurosurgery, Baylor College of Medicine, Houston, TX USA; 40 Department of Otolaryngology-Head and Neck Surgery, Baylor College of Medicine, Houston, TX USA; 41 Duncan Neurological Research Institute, Texas Children’s Hospital, Houston, TX USA; 42 Department of Molecular and Human Genetics, Baylor College of Medicine, Houston, TX USA; 43 Department of Radiation Oncology, Gangnam Severance Hospital, Yonsei University College of Medicine, Seoul, Republic of Korea; 44 Department of Neurosurgery, University of Naples Federico II, Naples, Italy; 45 Department of Diagnostics and Public Health, University of Verona, Verona, Italy; 46 Unit of Anatomic Pathology- Neuropathology, Fondazione IRCCS Istituto Neurologico Carlo Besta, Milan, Italy; 47 Instituto de Investigación Biomédica de Salamanca (IBSAL), University Hospital of Salamanca, Salamanca, Spain; 48 Department of Clinical Neuroscience, Karolinska Institutet, Stockholm, Sweden; 49 Department of Radiation Oncology, Faculty of Medicine and University Hospital Carl Gustav Carus, Technische Universität Dresden,, Dresden, Germany; 50 National Center for Tumor Diseases (NCT), Partner Site, Dresden, Germany; 51 German Cancer Research Center (DKFZ), Heidelberg and German Cancer Consortium (DKTK), Partner Site, Dresden, Germany; 52 OncoRay – National Center for Radiation Research in Oncology (NCRO), Faculty of Medicine and University Hospital Carl Gustav Carus, Technische Universität Dresden, Dresden, Germany; 53 Helmholtz-Zentrum Dresden – Rossendorf, Institute of Radiooncology, Dresden, Germany; 54 Department of Clinical Physiology and Nuclear Medicine, Copenhagen University Hospital-Rigshospitalet, Copenhagen, Denmark; 55 Department of Clinical Medicine, Faculty of Health and Medical Sciences, University of Copenhagen, Copenhagen, Denmark; 56 Department of Clinical Medicine and Biotech Research and Innovation Center (BRIC), University of Copenhagen, Copenhagen, Denmark; 57 Department of Congenital Disorders, Statens Serum Institut, Copenhagen, Denmark; 58 Department of Neurological Surgery, Istituto Nazionale Neurologico “C.Besta”, Milan, Italy

**Keywords:** Meningioma, Radiotherapy, Recurrence, Progression, Atypical meningioma, Resection

## Abstract

**Purpose:**

The role of adjuvant fractionated radiotherapy (aFRT) after gross total resection (GTR) of WHO-2 meningiomas remains unclear. We aimed to estimate the effect of aFRT on recurrence risk and survival following GTR and subtotal resection (STR).

**Methods:**

We analyzed 1452 patients with WHO-2 from our international, multicenter database (followed between 1989 and 2019). Outcomes were recurrence (10-year follow-up) and death (5-year follow-up). Risk estimates were obtained using competing risks and survival analysis. Average treatment effects were estimated by *G*-computation, adjusted for potential confounding by age, sex, Simpson grade, Ki-67 proliferation index, location, country group (universal healthcare or not), and year of treatment initiation. The robustness of findings was examined through sensitivity analyses.

**Results:**

Overall, 276 of 1452 patients (19.0%) received aFRT. Among GTR patients, unadjusted analysis showed comparable recurrence proportions between irradiated and non-irradiated patients (25.5% vs. 22.8% within 5 years). Adjusted analyses provided no evidence that aFRT reduced the risk of recurrence (largest difference: −2.7%, 95% CI −5.6 to 0.2); although, the CIs include the possibility of small beneficial effects. In STR patients, aFRT was associated with reduced recurrence risk in both unadjusted and adjusted analyses. Unexpectedly, a higher mortality was observed among irradiated GTR patients, largely driven by older patients with low Ki-67 PI receiving aFRT. Sensitivity analyses showed similar results for patients with STR but discrepancy in estimates for those with GTR.

**Conclusion:**

Adjuvant FRT showed a consistent reduction in recurrence risk after STR while inconsistent recurrence risk estimates were observed for patients with GTR. The findings reflect efficacy of aFRT using real-world data without standardized guidelines.

**Supplementary Information:**

The online version contains supplementary material available at 10.1007/s11060-025-05349-7.

## Background

While the primary treatment for WHO-2 meningioma is surgical resection, adjuvant fractionated radiotherapy (aFRT) continues to play a role in the management of patients. A subtotal resection (STR, Simpson grade 4) leaves a known residual that can be treated with aFRT or single fraction stereotactic radiosurgery (SRS) for local disease control [1]. However, evidence on the role of aFRT to prevent recurrence and improve overall survival following gross total resection (GTR; Simpson grade 1–3) to eradicate microscopic disease remains unclear [1]. Previous results have demonstrated a reduced risk of recurrence [2–6], while other studies have found no such difference between irradiated and non-irradiated patients [7–9]. The 5-year risk of recurrence for WHO-2 GTR patients receiving aFRT has been reported between 0% and up to approximately 50% [2, 4–6, 10], likely reflecting the heterogeneity of relatively small cohort sizes, different study designs, varying time periods, and variable follow-up.

Studies investigating the effect of aFRT on overall survival for patients with and without GTR are more limited. The largest retrospective cohort study to date, based on 2515 WHO-2 patients identified in *The National Cancer Database*, reported an overall survival benefit for STR patients who received aFRT. In contrast, no such benefit was observed for WHO-2 patients who underwent GTR and received aFRT [11]. These findings align with observations from other studies, which have similarly reported an overall survival benefit associated with aFRT in patients with STR [12–15]. Unfortunately, these studies did not consistently differentiate between aFRT and SRS and are either compiled from retrospective studies of variable quality or based on the Surveillance, Epidemiology, and End Results (SEER) database with known limitations related to radiation oncology.

Robust data from prospective trials is required to guide evidence-based decisions on whether to administer aFRT in the clinical management of WHO-2 meningiomas. Currently, the ROAM/EORTC-1308 (ISRCTN71502099) and NRG-BN003 (NCT03180268) trials are the only randomized controlled trials undertaken that randomize WHO-2 patients after GTR; the EORTC-1308 trial closed in 2021, with its results still pending, while the BN003 trial is expected to accrue patients until 2027 [16].

Using our international, multicenter database, our objectives were to (1) estimate and compare the risk of recurrence and death in WHO-2 meningioma patients either receiving FRT as an adjuvant treatment to GTR and STR, or not receiving aFRT, accounting for potential confounders; to (2) assess the robustness of these risk estimates; and to (3) examine trends in the usage of aFRT over the past two decades in published WHO-2 cohorts.

## Methods

### Database and study population

The *PERNS* (PERsonalized NeuroSurgery) database is a multicenter, international, retrospective database including adult patients who underwent resection of an intracranial meningioma. The database includes 7992 patients collected from 42 different institutions, who were diagnosed, surgically treated, and followed over a three-decade period from 1989 to 2019 [17]. Participating institutions contributed data collected from consecutive series of surgically treated meningioma patients. Data review was performed locally by representative physicians from various departments, including neurosurgery, neuropathology, neurology, and radiation oncology. Since data contributions were not collected in a uniform manner across centers, some variables were missing for certain sites.

### Variables to adjust for confounding and stratification

The following variables were included in the analyses: age, sex, WHO grade, Simpson grade, aFRT, Ki-67 PI, country, and year of treatment initiation. Simpson grade was determined intraoperatively by the neurosurgeon.

### Inclusion and exclusion criteria

**Fig. 1:**
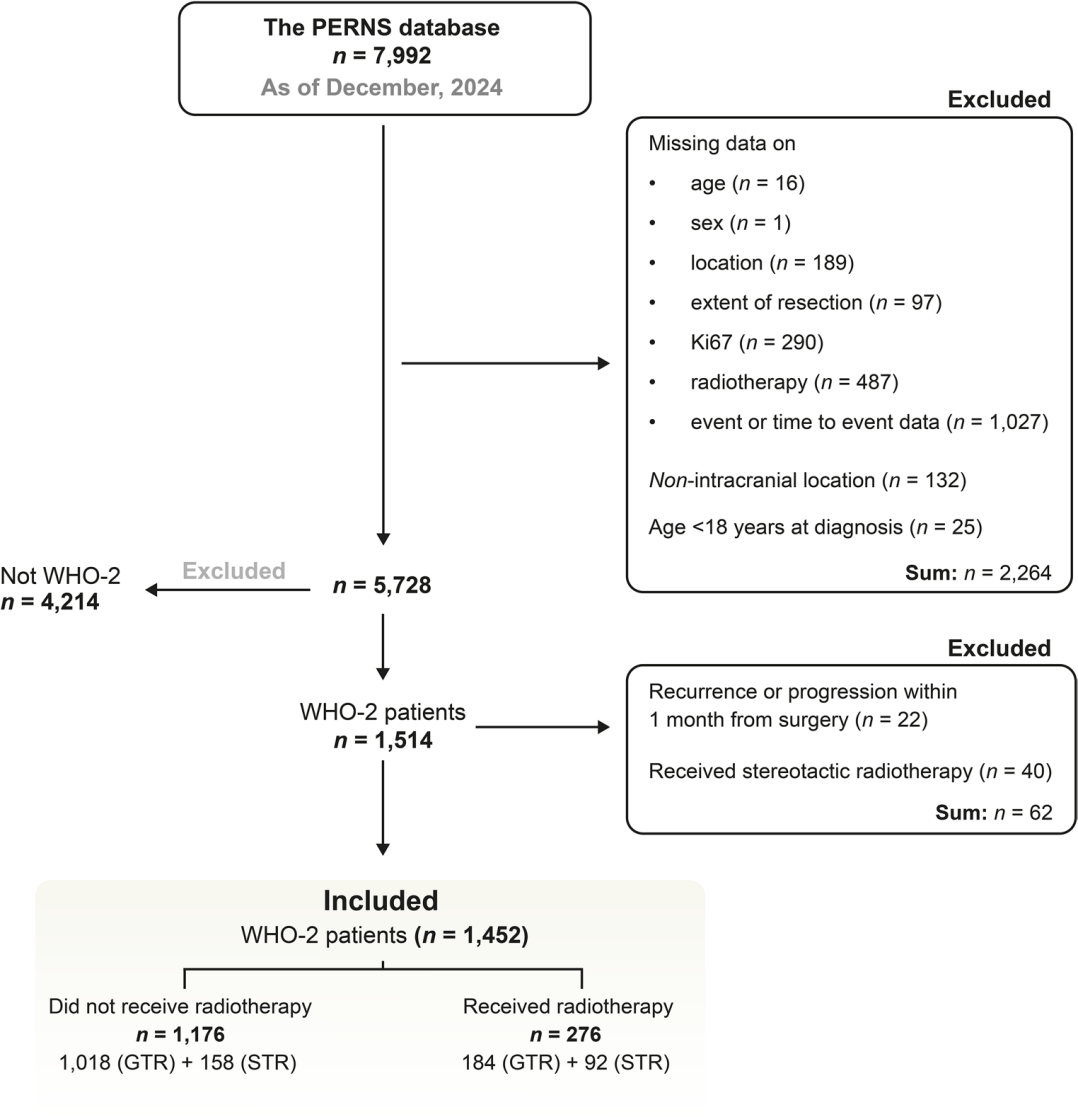
Flowchart over data inclusion and exclusion - from the *PERNS* database to the study population. Abbreviations: gross total resection (GTR), subtotal resection (STR)

Figure 1 delineates a flowchart of data exclusion and inclusion. In summary, patients included in the study cohort were older than 18 years, classified according to the 2007 or 2016 edition of the WHO classification, and had undergone resection of an intracranial WHO-2 meningioma. Patients with missing data on variables of interest were excluded as well as patients who received SRS, focusing exclusively on aFRT. In addition, patients who experienced recurrence or death within the first month could not be distinguished between those who had not yet started aFRT treatment and those who would not receive it and were therefore excluded.

### Exposure of interest

Patients who received aFRT following either GTR or STR were compared with those who did not receive this treatment following initial surgery of a primary WHO-2 meningioma.

### Outcomes of interest

Clinical endpoints included recurrence within 10 years postoperatively and death within 5 years, recognizing that mortality in this elderly population may be influenced by factors beyond aFRT and meningioma. Recurrence was defined by MRI or CT imaging where either increase in size of a known residual tumor or appearance of tumor growth after previous gross total removal was detected. Most data were accrued before publication of EANO guidelines or the introduction of standardized radiographic assessment criteria for meningiomas, hence the assessments were not standardized [1, 18]. However, all contributing centers are specialized, multidisciplinary institutions with systematic follow-up where new clinical symptoms would lead to scanning. Since aFRT is typically initiated weeks to months after surgery, patients receiving aFRT will by definition remain recurrence-free until treatment initiation. We acknowledge that this introduces immortal time bias during the early postoperative period; however, events occurring within this early period are rare and unlikely to substantially affect the results. Patients were censored alive and recurrence-free at the last recorded clinical follow-up if no event was observed.

### Statistical analysis

Death without a prior recurrence was considered a competing event, which was accounted for when analyzing the risk of recurrence [17]. We did not have information on toxicities related to aFRT.

The Aalen-Johansen method was used to describe the cumulative proportions (i.e., the unadjusted risks) of patients who had experienced a recurrence among those who received aFRT and those who did not [17]. In this analysis, patients were followed until 10 years postoperatively, and these results were generated for patients stratified based on the extent of resection (GTR vs STR). The Kaplan-Meier method was used for the analysis of death.

Regression standardization (also known as *G*-computation) was used to estimate the average risk of recurrence (and death) for a patient that *would* be randomly assigned to receive vs not to receive aFRT. It estimates what *could* be obtained in a randomized trial with an unadjusted risk analysis, where treatment assignment is independent of patient characteristics (when assuming that all potential confounders are adjusted for in the analysis) [19].

The risk difference between those who *would* receive aFRT and those who *would not* (with 95% confidence intervals [CI]) was reported at specified timepoints: 1.0-, 1.5-, 2.0-, 5.0-, and 10-years post-surgery. These were obtained from a multiple logistic regression model, which was fitted via inverse probability of censoring weighting [20], and adjusted for: aFRT (yes/no), age groups ( < 50 years, between 50 and 70 years, and > 70 years at diagnosis), intracranial location (skull base and non-skull base), sex, a country group with two levels based on universal health care coverage and accessibility (accounting for countries with different health-care systems; Group 1: Canada, Denmark, France, Germany, Hungary, Italy, Japan, Norway, South Korea, Spain, Sweden, and Switzerland. Group 2: Brazil, China, India, and USA), time period in which the patients were treated in ( < 2000, 2000 to 2007, > 2007) and Ki-67 PI group ( < 4, 4 to 8, > 8). Interaction terms were included to account for how the effect of aFRT may vary with (1) the Ki-67 PI and (2) the time period (of treatment initiation). The censoring weights were computed using the Kaplan-Meier method stratified on calendar year and whether aFRT was received.

### Sensitivity analysis

The average treatment effect was also estimated using propensity score weighting, which assigns weights to each individual based on the inverse probability of receiving aFRT given the covariates described above [20]. In contrast, *G*-computation (main analysis) models the outcome conditional on these covariates.

## Results

### Study cohort

The cohort included 1452 patients and was followed for 8379 person-years, with a median follow-up of 5.5 years (range: 0.1 to 22.8 years) [21]. During follow-up, 394 patients had a recurrence while 195 patients had died, of which 94 met this endpoint before having a recurrence. A total of 964 patients were censored alive and without a recurrence at the end of follow-up.

### Characterization of irradiated WHO-2 patients

In total, 276 out of 1452 patients received aFRT (19.0%). Patients with GTR who received aFRT had a higher Ki-67 PI (median of 10%) compared with those who did not receive aFRT (median of 7%), suggesting that more aggressive features led to referral for adjuvant treatment (Table 1). In addition, patients receiving aFRT were also slightly younger (median 55 vs. 60 years). In contrast, for patients with STR, Ki-67 PI levels were similar between the irradiated and non-irradiated patients, while patients selected for aFRT were younger with less than 13% (vs. 24%) being older than 70 years (Table 1).

**Table 1:** Clinical characteristics, demographics, and covariate distribution between non-irradiated and irradiated patients with GTR or str

	Gross Total Resection *n* = 1,202		Subtotal Resection *n* = 250	
	No aFRT (*n* = 1,018)	Received aFRT (*n* = 184)	No aFRT (*n* = 158)	Received aFRT (*n* = 92)
**Age group**				
**Median, IQR**	60 (49, 69)	55 (47, 62)	59 (47, 68)	57 (48, 65)
< 50 years	261 (26%)	62 (34%)	46 (29%)	30 (33%)
50 to 70 years	535 (53%)	113 (61%)	74 (47%)	50 (54%)
> 70 years	222 (22%)	9 (5%)	38 (24%)	12 (13%)
**Sex**				
Female	605 (59%)	108 (59%)	95 (60%)	42 (46%)
Male	413 (41%)	76 (41%)	63 (40%)	50 (54%)
**Simpson grade**				
1	435 (43%)	48 (26%)	0 (0%)	0 (0%)
2	300 (29%)	54 (29%)	0 (0%)	0 (0%)
3	130 (13%)	22 (12%)	0 (0%)	0 (0%)
4	0 (0%)	0 (0%)	158 (100%)	92 (100%)
Reported as “GTR” *Simpson grade ≤3*	153 (15%)	60 (33%)	0 (0%)	0 (0%)
**Country group**				
Universal health care	777 (76%)	93 (51%)	110 (70%)	50 (54%)
No universal health care	241 (24%)	91 (49%)	48 (30%)	42 (46%)
**Year of treatment**				
< 2001	101 (10%)	5 (3%)	10 (6%)	7 (8%)
< 2007	193 (19%)	30 (16%)	30 (19%)	18 (20%)
≥2007	724 (71%)	149 (81%)	118 (75%)	67 (73%)
**Radiation dose in Gy** (Median, IQR*)	0 (0, 0)	58 (54, 60)	0 (0, 0)	58 (54, 60)
**Ki-67 PI** (Median, IQR*)	7 (5, 10)	10 (7, 13)	7 (4, 10)	8 (5, 11)
**Tumor location**				
Non-skull-base	741 (73%)	138 (75%)	93 (59%)	45 (49%)
Skull-base	277 (27%)	46 (25%)	65 (41%)	47 (51%)

*Abbreviations*. IQR: interquartile range denotes 1T^st^ a nd 3^rd^ quartile. GTR: gross total resection. STR: subtotal resection. Ki-67 PIs: Ki-67 proliferation index

The India cohort had the highest within-country proportion of aFRT-treated WHO-2 patients, with 68.1% (32 out of 47) of its cohort receiving aFRT (Supplementary Figure 1A). Other countries with a high proportion of aFRT use within their cohorts included Japan (35.7%), South Korea (35.3%), and the USA (30.9%). In contrast, cohorts from countries such as Brazil, France, and Sweden reported no cases of aFRT. Among all aFRT-treated patients in the entire study population (*n* = 276), the majority came from cohorts in South Korea (30.8%), followed by cohorts in China (18.5%), the USA (18.1%), and India (11.6%) (Supplementary Figure 1B).

### Recurrence and aFRT

**Fig. 2:**
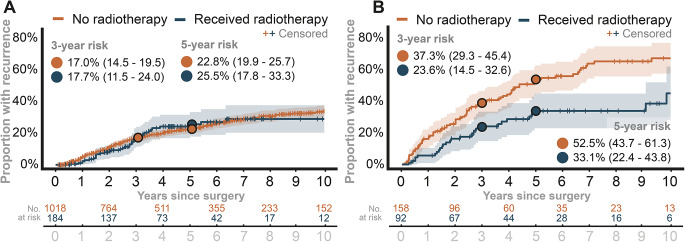
Comparison of proportions of patients who had a recurrence among those who received adjuvant fractionated radiotherapy to those who did not (using non-parametric Aalen-Johansen method). **A**: the proportion of recurrence among patients with gross total resection. **B**: the proportion of recurrence among patients with subtotal resection

For GTR patients, unadjusted analyses showed similar recurrence rates between the irradiated and non-irradiated groups. Within 5 years post-surgery, recurrence was observed in 22.8% (95% CI: 19.9 to 25.7) of patients without aFRT compared to 25.5% (95% CI: 17.8 to 33.3) of patients treated with aFRT (Fig. 2A). Among patients with STR who received aFRT, the proportion with recurrence was lower than in those not receiving aFRT. The 5-year recurrence proportions were 33.1% (95% CI, 22.4–43.8) vs 52.5% (95% CI, 43.7–61.3), respectively (Fig. 2B).

**Fig. 3:**
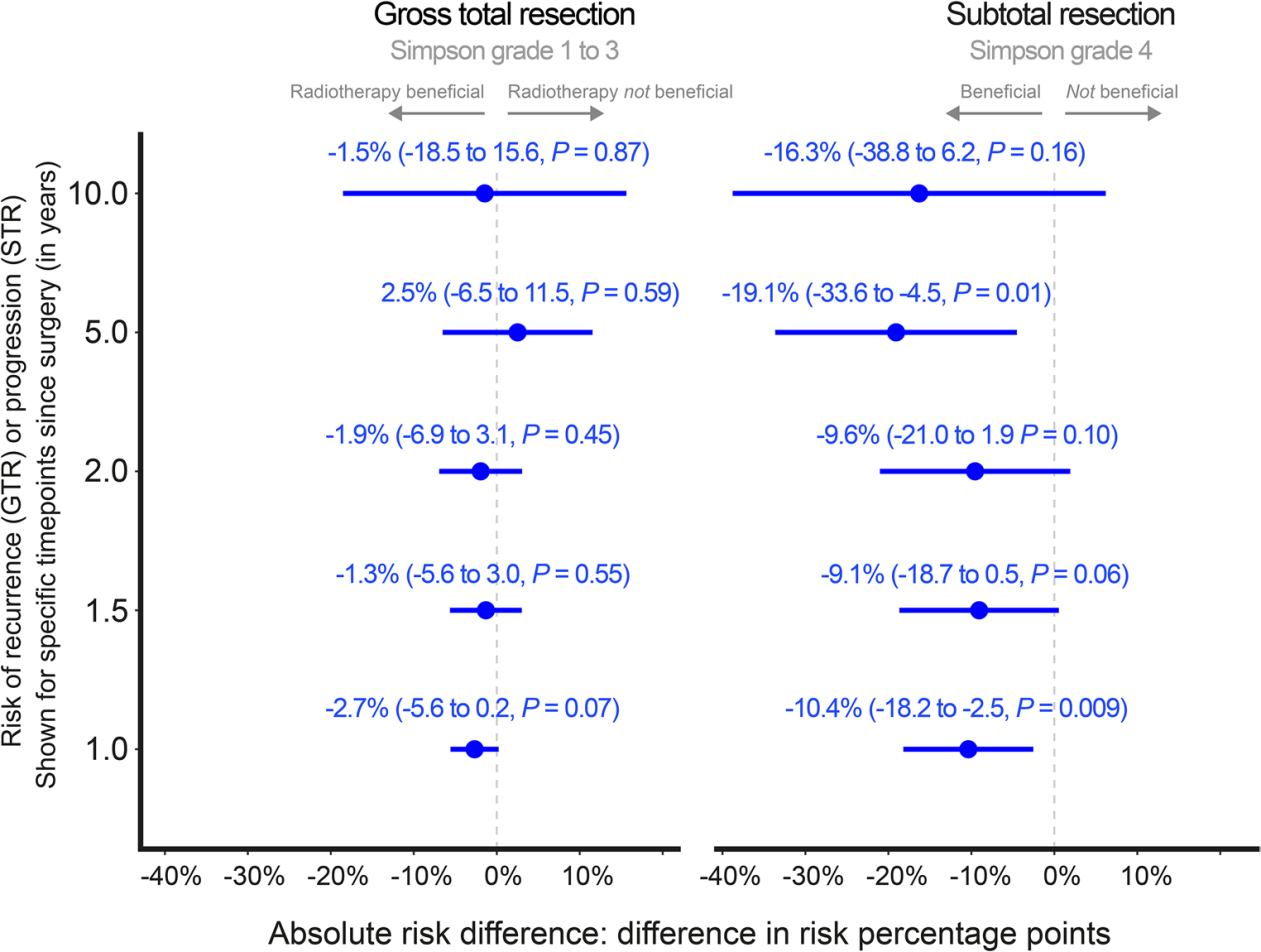
Risk of recurrence at the specified time points (y-axis). The estimated risk difference is the absolute difference between the risk for those who would receive adjuvant fractionated radiotherapy subtracted by the risk estimate for those who would not. In addition to the (absolute) risk difference is also the corresponding 95% confidence intervals and *P*-values. The risk estimate was obtained using standardization (*G*-computation) by utilizing logistic regression with right-censored data, fitted via inverse probability of censoring weighting

The main analysis, adjusted for confounders, showed that the difference in risk of recurrence was estimated close to 0% at all examined time points when comparing irradiated and non-irradiated patients with GTR. The largest estimated contrast was a −2.7% -point difference (95% CI: −5.6 to 0.2, *p* = 0.07) at 1-year post-surgery. Risk differences for GTR patients thus remained centered around zero with overlapping confidence intervals, indicating no measurable treatment effect within the observed period. For patients with STR, the risk difference for recurrence was statistically significant at both 1 and 5 years, ranging from −9.1% to −19.1%, indicating an early and sustained benefit of aFRT (Fig. 3). The widening of the confidence intervals over time likely reflects increasing uncertainty due to fewer patients remaining at risk during later follow-up.

### Sensitivity analysis using propensity score weighting

The resulting average treatment effect estimates were highly concordant with those from the main analysis, for both gross total and subtotal resections, supporting the robustness of the main findings to potential model misspecification due to unmeasured confounding (Supplementary Figure 2).

### Overall survival and aFRT

**Fig. 4:**
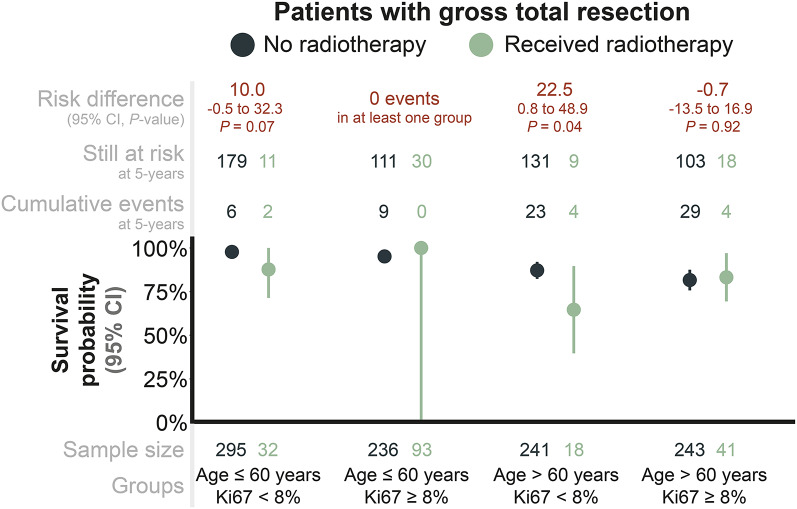
Unadjusted survival probability for patients with gross total resection when including data from South Korean cohorts, estimated using the Kaplan-Meier method

Based on the main analysis, the results indicated that GTR patients who *would* receive aFRT had a significantly higher 5-year risk of death compared to those who *would not* receive it, with a 10.3% risk difference (95% CI: 1.1 to 19.6, *p* = 0.03, Supplementary Figure 3). Further exploratory analysis was performed and included Kaplan-Meier analysis in subgroups defined by age groups (above vs below 60 years) and Ki-67 PI (above vs below 8%, the median). The subgroups indicated that the estimated effect was primarily driven by older GTR patients ( > 60 years) with *low* Ki-67 PIs ( < 8%) who had received aFRT (Fig. 4). As shown in Supplementary Figure 4, 50% of the older GTR patients with a Ki-67 PI below 8% and who received aFRT were from the South Korean cohorts. When data from the South Korean cohorts were excluded, the 5-year risk of death was similar between the irradiated and non-irradiated patient groups. These results could suggest that residual confounding exists, which may explain the unexpected 10.3% difference in 5-year survival probability presented above.

### Post-hoc analysis: robustness of the recurrence risk estimates

A sensitivity analysis was performed by excluding data from the South Korean cohorts in the recurrence risk analysis. This was motivated by (1) the above observation in the analysis of death and (2) the fact that approximately 31% of FRT cases were sourced from these cohorts, which is higher than for the other cohorts (Supplementary Figure 1B).

**Fig. 5:**
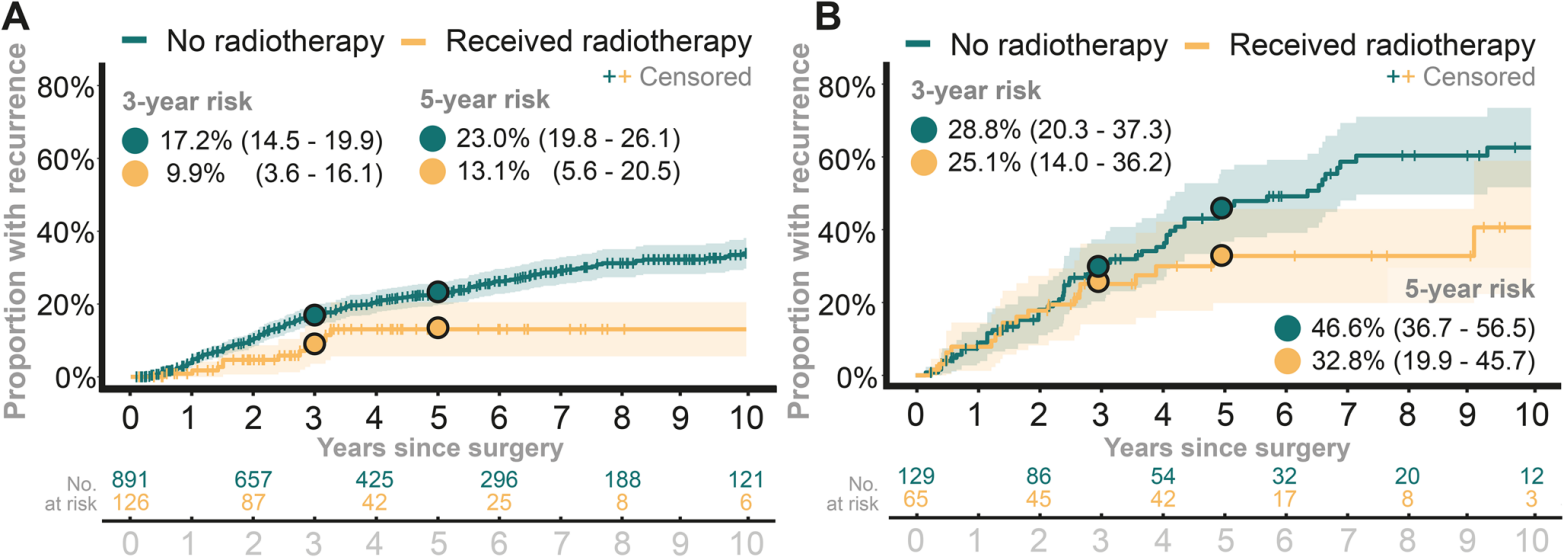
Sensitivity analysis exploring the robustness of observations presented in Fig. 2, but now excluding South Korean cohorts. Comparation of proportions of patients who had a recurrence among those who received adjuvant fractionated radiotherapy to those who did not (using non-parametric Aalen-Johansen method). **A**: The proportion of recurrence among patients with gross total resection. **B**: The proportion of recurrence among patients with subtotal resection

In this analysis and among GTR patients, the 5-year recurrence proportion was 23.0% (95% CI: 19.8 to 26.1) without aFRT and 13.1% (95% CI: 5.6 to 20.5) with aFRT (Fig. 5A). For STR patients, the proportion of recurrences remained consistent whether data from South Korean cohorts were included or excluded (with lower proportions seen in those receiving aFRT, Fig. 5B).

Supplementary Figure 5 presents re-analysis of the main results but with data from the South Korean cohorts excluded, now demonstrating a benefit of aFRT in GTR patients. For STR patients, the estimated short-term benefit of aFRT was no longer present. However, the estimated long-term benefit of aFRT remained significant regardless of whether data from the South Korean cohorts were included or excluded.

### Time trends associated with the application of aFRT

**Fig. 6:**
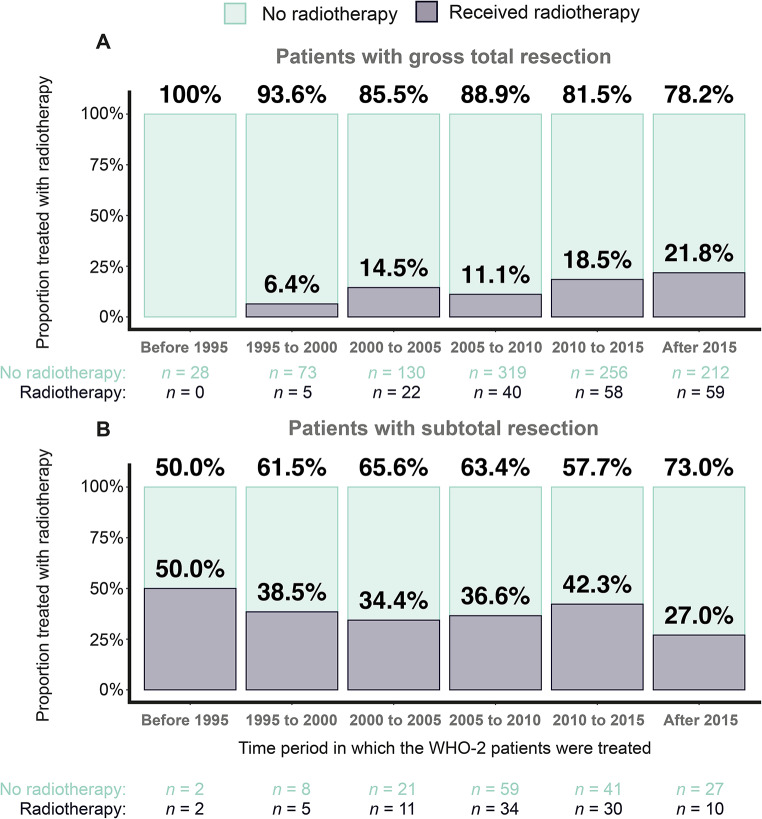
The proportion of patients receiving adjuvant fractionated radiotherapy or not for different time periods. **A**: Patients with gross total resection. **B**: Patients with subtotal resection

A gradual increase in the use of aFRT for patients with GTR throughout the study period was observed (Fig. 6A). Initially, none of the patients diagnosed before 1995 (*n* = 28) were treated with aFRT. This percentage increased to 6.4% for treatments given between 1995 and 2000 (*n* = 78), 14.5% for those treated between 2000 and 2005 (*n* = 152), and eventually reached 21.8% for patients treated after 2015 (*n* = 271) (Fig. 6A). In contrast, for patients with STR, no time trend was observed, with approximately 30% to 40% receiving aFRT during the study periods (Figure 6B).

## Discussion

No evidence of differences in meningioma recurrences were found between irradiated and non-irradiated patients with GTR when analyzing the entire cohort; however, these results were particularly sensitive to the composition of the data included. Subgroup analysis suggested that the untoward results were largely driven by outcomes in patients with lower Ki-67 PIs and age above 60.0 years (sourced from South Korean cohorts). The higher mortality observed among elderly GTR patients with low Ki-67 likely reflects multiple interacting factors rather than a direct treatment effect. Treatment-related toxicity may play an important role, as older patients are more susceptible to radiation-induced complications and its administration has been associated with negative survival benefits in this subgroup [22]. In addition, underlying frailty, comorbidities, and potential selection bias - where radiotherapy may have been offered to borderline-fit patients with limited expected benefit - could further contribute to this finding. When patients from these South Korean cohorts were excluded, a benefit for aFRT after GTR of WHO-2 meningiomas was observed. The only consistent finding across centers was long-term disease control for STR patients receiving aFRT compared to those who did not, which is in line with prospective data showing improved outcomes for patients receiving aFRT when undergoing a STR with a primary WHO-2 meningioma [10, 23]. The question of efficacy of aFRT for WHO-2 meningiomas undergoing GTR remains inconclusive in our analysis. Pooling large amounts of data from diverse centers could not resolve the existing uncertainty about the general efficacy of aFRT. Instead, the study demonstrated differences in aFRT effects in real-world settings without standardized protocols. Figure 6 further shows that time period alone appeared to influence aFRT use among GTR patients, underscoring temporal and institutional variability in practice. The findings can only reflect, that efficacy of aFRT must be evaluated in reference to the population treated. Indications for aFRT need to be refined since aFRT, like any treatment, can cause complications that must be balanced with potential benefit in each potential study-population.

### Regional differences and time trends

Previous studies indicate that aFRT for WHO-2 meningiomas is beneficial with STR, large size of tumor, younger age, and fewer comorbidities [5–7, 24, 25]. In this study, patients who received aFRT showed similar characteristics, as they typically had STR, were younger, and were diagnosed with higher Ki-67 PIs compared with patients not receiving aFRT. Obviously, radiation treatment was offered based on clinical deliberation, which differed between countries and centers. Cohorts from South Korea, China, and the USA accounted for 68% of the irradiated patients combined, while the non-irradiated patients were from other sites. Hence, identical patient conditions might lead to the administration of aFRT in cohorts from one country but not in another, indicating differences in practice. Additionally, patient factors and aFRT characteristics may differ regionally leading to differences in outcomes even after the same treatment paradigm, underscoring that estimates depend on the composition of the included data. These observations highlight the inherent heterogeneity of real-world data and the challenges it poses for estimating treatment effects across such diverse settings.

The proportion of GTR patients receiving aFRT increased from the earliest period (before 1995) to the most recent period (from 2015 onwards). The radiation technology advanced considerably over three decades such that it is possible that the reduction of radiation toxicities has made it more acceptable to offer aFRT to reduce recurrence risks. In contrast, the proportion of patients with STR receiving aFRT was relatively stable across the examined time periods, ranging between 30 and 40%. Regardless, time varying practices may contribute to inconsistencies in outcomes and require consideration when interpreting data across centers.

### Search of evidence-based clinical guidelines for aFRT in WHO-2 meningioma

Evidence from clinical trials on aFRT for WHO-2 meningiomas remains limited. The RTOG 0539 phase-II observational trial on intermediate-risk meningiomas (WHO-2 with GTR *or* WHO-1 with STR; *n* = 48) reported a 3-year progression-free survival of 93.8% following aFRT. However, merging these two clinically distinct groups, rather than differentiating between them, may be considered a limitation – especially with a relatively short-term endpoint (3 years), reflecting a follow-up during which most of these lesions do not typically recur [10]. Similarly, the EORTC 22,042–26042 phase-II observational trial reported a 3-year progression-free survival probability of 88.7% for WHO-2 with GTR (*n* = 56) after aFRT [26].

### Strength and limitation

The major strength of this study is its large sample size, which enables the exploration of potential differences at the individual institution level and the ability to compare variations across such different settings.

The primary limitation of our study is the potential selection bias, and the uncertain representativeness of the cohorts included in the *PERNS* database used to estimate the average causal effect of aFRT. The criteria for cohort selection or exclusion may not be consistently controlled as data contributions were compiled from multiple cohorts without a standardized inclusion framework. This heterogeneity limits our ability to ensure that the included cohorts comprehensively represent the global population of WHO-2 meningioma patients. Conversely, there are no evidence-based guidelines available, implying that”general representativeness” does not currently exist for WHO-2 patients receiving aFRT or not. As in many retrospective meningioma series, standardized radiographic criteria for recurrence were not available during most of the individual study periods [18]. While this is recognized as a limitation, it reflects the historical context of data acquisition precluding a unified retrospective meningioma series. However, all participating centers are specialized, multidisciplinary institutions with structured follow-up procedures, which ensued a certain level of clinical consistency. Finally, time to recurrence is defined by the first positive follow-up scan rather than when tumor regrowth biologically occurred. This inherent “interval bias” is an unavoidable limitation; moreover, we cannot exclude that variable intervals of follow-up may obfuscate precision of recurrence detection.

In addition, the lack of toxicity data limits interpretation of risk-benefit which is a key part of decision making for these patients. Finally, we lacked standardized information on radiation therapy details, such as fractionation scheme, target definition, or treatment modality (e.g., intensity-modulated radiotherapy [IMRT] vs. three-dimensional conformal radiotherapy [3D-CRT]), and of which may also have contributed to heterogeneity in treatment effects across centers and over time.

The diagnostic criteria for WHO-2 meningiomas have changed throughout the study period (from 2007 to 2016); however, a previous study showed that re-classification affects only 3.9% of WHO-1 patients due to the added brain invasion criterion, suggesting only a minor impact on the presented results [27].

Molecular subgroups and classifiers are increasingly considered in diagnostics with more consistent prognostication of meningiomas [28–32]. Molecular data were not available in this study, and the extent to which this may have influenced the results is unknown. Atypical WHO-2 meningiomas have previously been shown to segregate into “benign,” “intermediate,” and, less frequently, “malignant” methylation classes, reflecting the biological heterogeneity within this group [28]. Given these differences, variable responses to aFRT would be expected depending on molecular subgroup. Some of this heterogeneity may have been indirectly captured through adjustment for Ki-67 PI, which serves as a partial proxy for underlying molecular properties. Nevertheless, the inability to fully account for molecular features represents a limitation of this study. This consideration further extends to recent updates (cIMPACT-NOW 8), where meningiomas with WHO-1 morphology but with chromosome *1p* deletion together with *22q* deletion and/or an *NF2* oncogenic variant should be assigned WHO-2. Consequently, a proportion of meningiomas previously classified as WHO-1 would now be reclassified as grade 2 and is similarly considered a limitation [33, 34].

## Conclusion

Among patients with WHO-2 meningiomas with GTR, our large multicenter dataset did not find robust evidence of clinical benefit of aFRT. The only consistent finding was long-term disease control of STR following aFRT. Elderly patients with GTR and less aggressive lesions may experience more risks than overall survival benefits from aFRT. The analysis of WHO-2 meningiomas within the *PERNS* database demonstrated considerable heterogeneity, likely reflecting differences in treatment practices, and highlighting the need of standardizing aFRT protocols to target populations that benefit most. These findings underscore the importance of prospective evidence and molecularly guided stratification.

## Electronic supplementary material

Below is the link to the electronic supplementary material.

Supplementary Material 1
