# Supplementary material for: Fractionated radiotherapy adjuvant to surgery of WHO-2 meningioma with and without gross total resection: a multicenter, retrospective cohort study of 1,452 patients
Source: J Neurooncol. 2026 Feb 9;176(3):201. doi: 10.1007/s11060-025-05349-7 (PMC12886253; doi:10.1007/s11060-025-05349-7)
Supplement: Supplementary file 1 — Supplementary Material 1 [file 11060_2025_5349_MOESM1_ESM.docx]

**Supplementary Figure 1. A:** The distribution of total patients receiving aFRT (*n* = 276) within each local series from where data were sourced. **B:** aFRT patient proportion per each countries respect the whole present series analyzed

**Supplementary Figure 2.** This figure reproduces the result of **Figure 3** (results of the main analysis) and additionally presents the results using propensity score weighting (results of the sensitivity analysis). This is to provide a head-to-head comparison when estimating the average treatment effect when modeling the outcome based on covariates (*G*-computation in the main analysis) or using the same covariate instead to propensity score weight each individual based on the probability of receiving aFRT.

**Supplementary Figure 3.** The average treatment effect between irradiated and non-irradiated patients with gross total resection using death as outcome (*G*-computation).

**Supplementary Figure 4**. The distribution of patients receiving aFRT while older than 60 years and with a Ki-67 PIs below 8% is shown (*n* = 18) based on the country from where the data were sourced.

**Supplementary Figure 5.** Sensitivity analysis. This figure reproduces the result of **Figure 3** (results of the main analysis) and additionally present the results using the same method after excluding data from South Korean cohorts (results of the sensitivity analysis). This is to provide a head-to-head comparison and it summarizes the sensitivity of the results to the data from South Korean cohorts.
